# Propofol lipidic infusion promotes resistance to antifungals by reducing drug input into the fungal cell

**DOI:** 10.1186/1471-2180-8-9

**Published:** 2008-01-17

**Authors:** Sofia Costa-de-Oliveira, Ricardo Araujo, Ana Silva-Dias, Cidália Pina-Vaz, Acácio Gonçalves Rodrigues

**Affiliations:** 1 Department of Microbiology, Faculty of Medicine, University of Porto, Porto, Portugal; 2 Department of Microbiology, Hospital S. João, Porto, Portugal; 3 Burn Unit, Department of Plastic and Reconstructive Surgery, Faculty of Medicine, University of Porto and Hospital S. João, Alameda Prof. Hernani Monteiro, 4200, Porto, Portugal

## Abstract

**Background:**

The administration of non-antifungal drugs during patient hospitalization might be responsible for discrepancies between *in vitro *and *in vivo *susceptibility to antifungals. Propofol is often administered to intensive care units as a sedative.

The purpose of this study was to evaluate the effect of propofol lipidic infusion upon the growth and susceptibility profile of pathogenic fungi.

*Candida *and *Aspergillus *were studied regarding the ability to grow and its susceptibility profile to antifungals in the presence of propofol infusion (Fresenius^®^) (1.25, 2.5 and 5 mg.ml^-1^) and its lipidic vehicle. The intensity of fluorescence after staining with FUN1, in the presence and absence of propofol infusion, was determined by flow cytometry. Radioactivity assays were also performed in order to quantify the input of [^3^H]- itraconazole into the fungal cell in the presence of propofol. Assays were repeated after addition of sodium azide, in order to block efflux pumps.

**Results:**

Propofol infusion promoted budding of *Candida *and the germination of *Aspergillus*, latter forming a lipid layer around the hypha. An increase of minimal fungicidal concentrations regarding both *Candida *and *Aspergillus *strains was found for all antifungals when incubated simultaneously with propofol infusion. A decrease of the intensity of fluorescence of *Candida *cells was systematically observed, as well as a significant reduced intracellular uptake of [^3^H] itraconazole in cells treated with propofol infusion, even after the blockade of efflux pumps. The results obtained when testing with the lipid vehicle were similar.

**Conclusion:**

Propofol infusion, due to its lipidic vehicle, increased the fungal germination and promoted resistance to antifungals. This effect seems to be related to the reduced access and/or permeabilization to fungal cells by antifungals.

## Background

Discrepancies between *in vivo *and *in vitro *susceptibility to antifungals discourage microbiologists and clinicians regarding the routine use of susceptibility testing methods. Although *in vitro *resistance usually correlates with clinical resistance, high susceptibility *in vitro *is not always related to clinical success, particularly for *Aspergillus *spp., most strains being susceptible *in vitro *[1]. Ultimately, the mortality rates are unacceptably high in patients treated with antifungals that showed high *in vitro *efficacy [1,2]. Propofol is an intravenous hypnotic agent very popular for induction and maintenance of general and intravenous anaesthesia. It is commonly administered in Intensive Care Units to critically ill patients, often under mechanical ventilation, which represent a high risk group for health care related infections. The use of propofol has been previously associated to an increased risk for infection, although some controversy still remains [3,4]. It was proposed a low risk of contamination whenever providing standard hygienic precautions [3,5]. Nevertheless, other observations described the lipid emulsion of propofol as a good culture medium to support the growth of *Candida albicans *and *Escherichia coli *[6,7]. Additionally, other reports associated post surgical infections with the extrinsically contamination of propofol infusion [5,8]. Propofol has also been shown to inhibit a variety of functions of neutrophils *in vitro*, although such effect was not so evident *in vivo *[9].

We have studied the effect of the infusion of propofol and its lipidic vehicle upon antifungal susceptibility of *Candida *and *Aspergillus *spp. A promotion of resistance due to a decreased input of the antifungal drugs was found.

## Results

**Figure 1:**
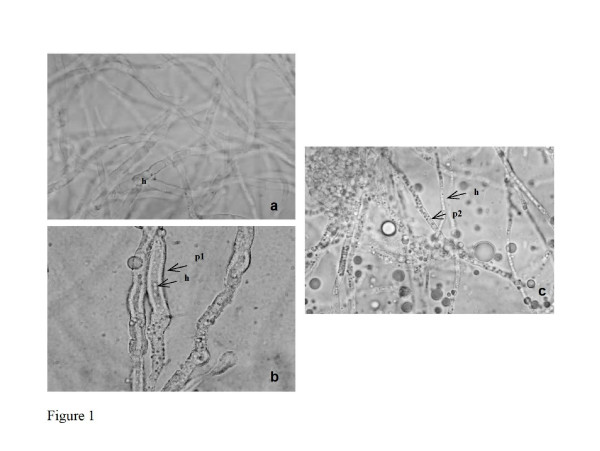
Representative example of *Aspergillus fumigatus*, after 24 hours of incubation: a. non-treated cells; b. cells treated with 5 mg.ml^-1 ^of propofol infusion; c. cells treated with 5 mg.ml^-1 ^of propofol infusion and washed thrice in sterilized water. (**h **hypha; **p1 **lipidic layer around hypha; **p2 **lipidic drops).

In *Candida *strains, budding and germ tube formation were similar in presence of all tested concentrations of propofol infusion. In non-*albicans *strains, the incubation with 5 μg.ml^-1 ^of propofol infusion resulted in a significant increased of cells with buds when comparing to control (71.5% ± 7.46 *versus *26.6% ± 4.15, *C. parapsilosis n *= 5 as a representative example) (*p *< 0.001). Conversely, a significant reduction of germ tube formation was observed in *C. albicans *strains comparing with non-treated yeasts (17.3% ± 6.29, *versus *76.2% ± 8.69, *n *= 5) (*p *< 0.001). *Aspergillus *conidia germinated in RPMI 1640 culture medium after 4 to 6 hours, the hyphal form being obtained following 10 to 12 hours. Propofol infusion supported the germination of all *Aspergillus *strains, showing similar values to those obtained in plain RPMI 1640 medium. A significantly higher percentage of germination of *A. fumigatus *conidia was found in PBS plus propofol and plain propofol infusion in comparison with plain PBS (21% ± 3.6 *versus *6% ± 1.6, *n *= 5) (*p *= 0.047) following 6 hours at 37°C. Longer incubation periods of conidia with propofol infusion showed lipidic drops or a lipidic layer around the hypha, in all assays and with all strains, which remained present following several washings steps (Figure 1).

The result of MIC determination revealed that all fungal strains were susceptible to the tested antifungals. Propofol infusion or its vehicle, at the tested concentrations, consistently promoted an increase of MFC mean values for *Candida *and *Aspergillus *strains(Table 1), this effect being dose-dependent and statistically significant (*p *< 0.001); such effect was invariably observed with all strains of *Candida *and *Aspergillus *and with all antifungals, in some cases the mean values increasing over 4 fold. MFC values in the presence of 5 mg.ml^-1 ^of propofol infusion or its vehicle increased at least 2 dilutions in all strains (above the error rate of the method) for fluconazole and voriconazole, more than 3 dilutions for amphotericin B and 4 to 5 dilutions for posaconazole and itraconazole. There was no large variability between the tested strains.

**Table 1:** Minimal fungicidal concentration (MFC) values of *Candida*and *Aspergillus *strains to AMB (amphotericin B), FLC (fluconazole), ITC (itraconazole), VRC (voriconazole) and PSC (posaconazole), determined by CLSI protocols, in the absence and presence of propofol infusion

		Strains (n)						
		
		MFC values* range μg.ml^-1^						
		
Antifungals	Propofol(mg.ml^-1^)	*C. albicans *(5)	*C. tropicalis *(5)	*C. parapsilosis *(5)	*C. glabrata *(5)	*A. fumigatus *(5)	*A. flavus *(4)	*A. niger *(4)
AMB	0	0.25	0.25–1	0.06–0.125	0.25–0.5	0.25–0.5	0.5–1	0.06–0.25
	1.25	0.5–1	1	2	2	1–4	1–>16	0.25–1
	2.5	0.5–2	1–2	2	2	2–4	1–>16	0.25–1
	5	2–4	2	2–8	4–8	4–16	4–>16	0.5–2

FLC	0	8–16	4–8	8–16	16	nd	nd	nd
	1.25	16–>64	>64	>64	>64	nd	nd	nd
	2.5	16–>64	>64	>64	>64	nd	nd	nd
	5	16–>64	>64	>64	>64	nd	nd	nd

ITC	0	nd	nd	nd	nd	0.5–16	0.125–0.25	0.125–16
	1.25	nd	nd	nd	nd	>16	2–>16	>16
	2.5	nd	nd	nd	nd	>16	8–>16	>16
	5	nd	nd	nd	nd	>16	16–>16	>16

VRC	0	0.06–0.25	0.25–0.5	0.5–1	4–8	0.25–4	0.25–2	0.06–8
	1.25	0.25–>2	>2	>2	>8	2–8	0.25–2	1–16
	2.5	0.5–>2	>2	>2	>8	2–8	0.5–4	1–16
	5	1–>2	>2	>2	>8	2–8	0.5–4	1–16

PSC	0	0.06–0.125	0.03–0.125	0.06–0.125	1–2	0.03–0.25	0.03–0.06	0.06–1
	1.25	1–2	>2	2	2	1–4	0.25–2	0.125–8
	2.5	>2	>2	2	>2	1–4	0.5–2	0.25–16
	5	>2	>2	>2	>2	1–4	0.5–4	0.25–>16

^nd ^not done

* The mean value for each strain was considered

**Figure 2:**
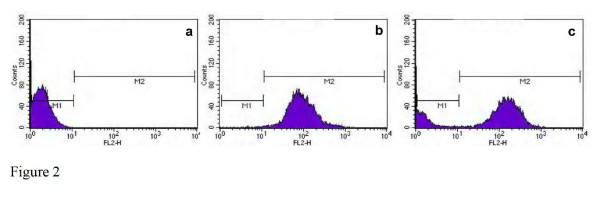
Flow cytometric histograms representing the emitted fluorescence after 90 minutes by: a. non-stained yeast cells (autofluorescence); b. cells treated with sodium azide and stained with FUN1; c. cells treated with sodium azide and 5 mg.ml^-1 ^of propofol infusion and stained with FUN1 (strain of *Candida albicans *shown as a representative example).

Flow cytometry analysis of *Candida *blastoconidia resulted in 98% of cells stained with FUN1, even after the incubation of sodium azide (Figure 2b). However, after treatment with propofol infusion and stained with FUN1, a non-stained sub-population of cells, similar to autofluorescence, of around 15% was revealed soon after 90 minutes (Figure 2c). This fact was unrelated with the incubation with sodium azide at a concentration able to block the efflux pumps (Figure 2c). The azole resistant strain of *C. albicans *95–190 (with overexpression of efflux pumps genes), after treatment with propofol infusion and stained with FUN1, revealed a non-stained sub-population, similar to other susceptible strains, even after the blockade of the efflux pumps with sodium azide. This effect was similar in presence of all the tested concentrations of propofol.

**Figure 3:**
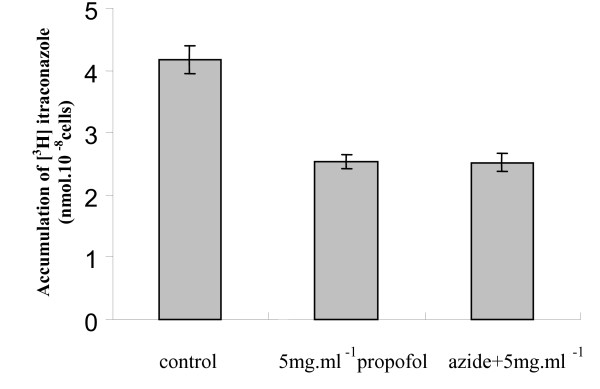
Effect of propofol up on [^3^H]-labelled itraconazole accumulation in antifungal susceptible strain *C. albicans *ATCC 90028. The accumulation of itraconazole was measured in the absence and presence of 5 mg.ml^-1 ^of propofol and after a prior incubation with 0.1 mM sodium azide. Dispersion bars relates to standard deviation.

After 1 hour, the accumulation of [^3^H]-labelled itraconazole was detected in blastoconidia cells (control cells) (Figure 3). However, a decrease of 39% in intracellular [^3^H]-labelled itraconazole was seen when yeast cells were incubated with propofol lipid infusion (Figure 3). This effect was similar in presence of the different tested concentrations of propofol lipidic infusion and did not increase following the pre-incubation with sodium azide (Figure 3).

The results obtained in the different assays were similar when performed with propofol lipidic vehicle.

## Discussion

Propofol administered in a lipid-based emulsion to patients has been shown to represent an excellent vehicle for supporting the growth of various microorganisms [6,7]. In the present study we clearly showed that budding of *Candida *spp. was promoted when incubated in the presence of propofol infusion, significantly more than in a culture medium. *Aspergillus fumigatus *germinate faster than the other *Aspergillus *species, possibly being associated to higher pathogenicity [10]. We showed that propofol infusion also supported mould germination and growth, apart from yeasts. It is important to emphasize that the propofol concentrations used in all assays are within the plasma levels often achieved in clinical practice [11,12].

Due to the opacity of propofol infusion or its lipidic vehicle it was not possible to determine MIC values. As the conventional susceptibility phenotypes refer to MIC values, we could not establish the corresponding phenotypes after incubation with propofol. In the case of amphotericin B (a fungicidal drug to both *Candida *and *Aspergillus*) MFC, determined after culture in agar solid medium, usually corresponds to MIC values; for the other antifungals, MFC values are usually located within one or three dilutions above the MIC. Some *C. albicans *and *C. tropicalis *showed trailing phenotype (about 2 dilutions) for azoles. This common effect may influence the evaluation of MFC values. However, trailing is only azole dependent and was not affected by the presence of propofol infusion. Nevertheless, we could observe a significant promotion of the MFC value in presence of propofol infusion, in some cases up to 4 dilutions, suggesting that the strains became quite tolerant to the effect of all antifungals. FUN1 is a fluorescent probe that is converted by metabolically active yeasts in intracytoplasmic vacuolar structures [13]. We have used this probe to study susceptibility of yeasts after incubation during one to two-hours with the antifungals; a higher intensity of fluorescent in susceptible strains, decreasing in resistant strains was described [14,15]. The decrease of the intensity of fluorescence could be explained by the presence of energy-dependent efflux pumps, which were reverted with sodium azide or several modulators [13,14]. In the present study, the viable cells showed a decrease of the intensity of fluorescence in the presence of propofol which was not however reverted with sodium azide, even considering the resistant tested strain. Furthermore, cells were washed several times before the addition of the fluorescent probe in order to avoid its binding to propofol infusion. These results support that propofol infusion affected FUN1 access and/or permeabilization, reducing the input of the fluorescent probe into the cell. We had also previously shown that [^3^H]-labelled itraconazole accumulation could be a useful tool to confirm the existence of efflux pumps in resistant *Candida *strains to azoles [16]. The blockade of efflux pumps with sodium azide resulted in the reduction of input of [^3^H]-labelled itraconazole in *Candida *cells, after incubation with propofol infusion. In fact, propofol infusion was responsible for the difficult access of itraconazole to the fungal cell.

Attending to the facts that i) the increase of MFC values was similar with all antifungal drugs, including the recently available posaconazole; ii) FUN1 could not stain a considerable percentage of the yeast cells in presence of propofol infusion and iii) a lesser amount of [^3^H]-labelled itraconazole was present within such cells, even after the addition of sodium azide, we are forced to conclude that the promotion of antifungal resistance in presence of propofol lipidic infusion results from a reduced access and/or permeabilization of antifungal agents into the fungal cells. The same results were obtained when only the propofol vehicle was used, thus we had concluded that the major responsible for such effect was the lipidic nature of propofol infusion. The formation of a lipidic layer surrounding the cells supports such assumption, being responsible for the reduced permeabilization. Several studies reported that the lipidic membrane of fungal cells plays an important role in susceptibility to azoles [17,18]. Fluctuations in the lipidic environment affects, not only drug diffusion but also efflux pumps, coded specially by Cdr1 and Pdr5p, leading to multidrug azole resistance [19,20]. In our study, as the efflux pumps were blocked with sodium azide, the increased MFC values probably resulted from poor diffusion of the antifungal drugs caused by the lipidic vehicle layer or drops deposited around the cells. The hypothesis that propofol could be binding the antifungal agent in culture medium reducing the free number of molecules available for penetrating the cells was also raised. MFC results were more evident when propofol was associated with itraconazole or posaconazole (increase of 4 to 5 dilutions), both lipophilic drugs. However, we must consider that different concentrations of propofol resulted in similar effects. The hypothesis that the lipidic vehicle of propofol might be playing a role in sterol homeostasis and changing the azole target, should be considered attending to other research [21]. Lipid uptake by the fungal cell was described in the presence of the azoles following longer incubation time (overnight) [21]. Measuring sterols in azole-treated fungi in the presence or not of the lipidic vehicle of propofol could be interesting however, the incubation time with propofol on cytometric and radioactivity studies was very short to allow the incorporation of the lipid in membrane.

## Conclusion

The assays described in this manuscript provided an opportunity to describe the effect of propofol infusion in antifungal drug resistance. We concluded that propofol infusion, due to its lipidic vehicle reduced the access and/or permeabilization to *Candida *and *Aspergillus *cells to main antifungals administered to patients.

The described effect should raise the alert to a promoted risk of fungal infections in patients receiving propofol infusions, resulting from the fact that fungal strains become increasingly resistant to antifungals.

## Methods

### Strains

Twenty clinical strains of *Candida *spp. (5 *C. albicans*, 5 *C. tropicalis*, 5 *C. glabrata *and 5 *C. parapsilosis*) and thirteen strains of *Aspergillus *spp.(5 *A. fumigatus*, 4 *A. flavus *and 4 *A. niger) *were studied. *C. albicans *95–190, resistant to azoles by overexpression of efflux pumps genes (CDR1 and CDR2), was used during cytometric approach (strain kindly gift by Prof. Theodore White). Until testing, yeasts and moulds were kept frozen in Brain-Heart broth (Difco Laboratories, Detroit, MI, USA) with 5% glycerol. For each experiment, the strains were subcultured twice on Sabouraud agar (Difco) at 35°C, 48 hours for *Candida *and 7 days for *Aspergillus*.

### Drugs and Chemicals

Propofol infusion Fresenius^® ^(Kabi, France) at stock concentration of 1% was used. Propofol vehicle (soya bean oil, egg lecithin, glycerol, sodium hydroxide and sterile water) was also assayed. Fluconazole and voriconazole were obtained from Pfizer (Groton, CT, USA), amphotericin B from Bristol-Myers Squibb (New York, USA), itraconazole from Janssen-Cilag (Beerse, Belgium) and posaconazole from Shering-Plough (Kenilworth, NJ, USA). Antifungals drugs were maintained in stock solution at -70°C until use. [^3^H]-labelled itraconazole was supplied by Janssen-Cilag. Sodium azide was purchased from Sigma (Sigma-Aldrich, Germany).

### Growth assays

After cultivation of *Candida *and *Aspergillus *strains in Sabouraud agar medium (Difco, Detroit, MI, USA), a 5 × 10^6^.ml^-1 ^blastoconidia or conidia suspension of *Candida *and *Aspergillus *was prepared in phosphate buffer saline (PBS) (Sigma) and 100 μl were added in two parallel serial dilutions of propofol infusion (stock solution at 1%) and its vehicle (both at 0, 1.25, 2.5 and 5 mg.ml^-1 ^final concentrations) in RPMI 1640 culture medium (Sigma), PBS and plain propofol infusion in a final volume of 500 μl. RPMI is a hydrophilic medium, however, solubility problems were not found. For *Candida *strains, samples were collected after 3 hours incubation at 37°C, the cells were observed under phase contrast microscopy (Leitz Larborlux K) and the percentage of budding and germ tube formation (for *C. albicans*) were determined [22]. For *Aspergillus*, the percentage of conidial germination was determined after incubation for 6, 12 and 24 hours at 37°C [10].

### Susceptibility testing

For *Candida *spp., the minimal inhibitory concentration (MIC) to fluconazole, voriconazole, posaconazole and amphotericin B (tested concentration range: 0.125–64 μg.ml^-1^, 0.03–16 μg.ml^-1^, 0.03–16 μg.ml^-1 ^and 0.03–16 μg.ml^-1^, respectively) were determined accordingly the CLSI protocols M27-A2 (formerly NCCLS) [23]. Strains were classified as susceptible (S), susceptible-dose dependent (S-DD) and resistant (R) to fluconazole according to breakpoints defined by CLSI [23]. For voriconazole MICs ≤ 1 μg.ml^-1 ^were considered S, MIC = 2 μg.ml^-1 ^considered S-DD and MIC ≥ 4 μg.ml^-1 ^considered R [24]. Although susceptibility breakpoints have not yet been established for amphotericin B and posaconazole, strains with MIC ≤ 1 μg.ml^-1 ^were considered susceptible [25,26]. Minimal fungicidal concentration (MFC) to all antifungals was also determined. The content of each well containing drug concentrations to and higher than the MIC, and also the positive growth control were transferred to Sabouraud dextrose agar plates and incubated at 35°C for 48 h, as previously described [27]. The MFC was the lowest drug concentration that killed ≥ 99% of the final inoculum.

For *Aspergillus *spp., the CLSI protocol M 38-A was used to determine MIC values of voriconazole, posaconazole, itraconazole and amphotericin B (all antifungals tested concentration ranged 0.03–16 μg.ml^-1^) [28]. Although the unavailability of breakpoints for *Aspergillus *species, we followed several researchers that consider values of MIC ≤ 1 μg.ml^-1 ^as susceptible [29,30]. MFC values were determined, as previously described [31], and defined as the lowest drug concentration that showed either no growth or fewer than three colonies to obtain approximately 99 to 99.5% killing activity.

The susceptibility tests to the antifungals mentioned above were repeated in the presence of the propofol infusion or its vehicle in three distinct concentrations (1.25, 2.5 and 5 mg.ml^-1^). Since propofol infusion and its vehicle are opaque solutions, making impossible MIC determination, the content of each well containing antifungal + propofol drugs was cultured for MFC determination and values compared with the MFC to antifungals alone.

### Flow cytometry analysis

Yeast cells were incubated at 150 rpm, overnight, until late exponential growth, in Sabouraud broth (Difco) at 37°C. Yeasts cells were harvested after centrifugation and a 1 × 10^6 ^cells.ml^-1 ^suspension was prepared in PBS supplemented with 2% glucose (GH solution) and later divided into aliquots of 1 ml. The cells were then incubated with different concentrations of propofol infusion (0, 1.25, 2.5 and 5 mg.ml^-1^) at 37°C for 90 minutes and afterwards washed thrice, ressuspended in sterile water supplemented with 2% glucose and stained with 0.5 μM FUN1 (Molecular Probes, Europe BV, Leiden, Holland) for 30 minutes at 37°C. A Beckman Coulter XL-MCL flow cytometer (Beckman-Coulter Corp., Hialeah, FL, USA) equipped with a 15 nm argon laser was used. From each suspension 30000–50000 cells were analysed. The intensity of fluorescence emitted by cells treated with propofol infusion was determined at FL2 (575 nm) and compared with non-treated cells (control). In parallel experiments, yeast cells were treated with 0.1 mM sodium azide during 30 minutes, prior to incubation with propofol, as previously described [16], in order to block efflux pumps; thereafter, the same flow cytometry analytical protocol was used.

### Intracellular accumulation of [^3^H]-labelled itraconazole

*Candida *cells were initially incubated under similar conditions as previously described for flow cytometry assays. The cells were harvested by centrifugation at 5000 rpm for 10 minutes at 4°C, washed thrice, ressuspended in PBS at a final concentration of 2.5 × 10^8 ^cells.ml^-1 ^and incubated with 0, 1.25 and 5 mg.ml^-1 ^of propofol infusion at 37°C, with continuous shaking at 300 rpm for 30 minutes [16]. Parallel experiments were prepared, but also involving a pre-incubation of the yeasts cells with sodium azide at 0.1 mM. The cells were washed thrice and [^3^H]-labelled itraconazole was added to yeast suspensions at a final concentration of 3 μM, as previously described[16]; the cells were incubated in glass vials at 37°C, with continuous shaking (300 rpm), during 1 hour and then harvested by centrifugation at 5000 rpm for 10 min at 4°C, washed thrice with 3 ml of ice-cold PBS containing 10 μM unlabelled itraconazole. The pellets were later ressuspended in 500 μl of PBS and the radioactivity was determined, following the addition of a scintillation cocktail (Optiphase "Hiphase3", Perkin-Elmer), in a liquid scintillation counter (LKB Wallac, 1209 RackBeta).

### Lipidic vehicle experiments

All the described experiments were repeated in the presence of the propofol lipidic vehicle used in Fresenius^® ^formulation (soya bean oil, egg lecithin, glycerol, sodium hydroxide and sterile water).

### Statistical analysis

The effects of different concentrations of propofol upon germination of fungal cells and MFC values of the distinct antifungals were compared using one-way analysis of variance (ANOVA) and Student's *t*-test. Significance was accepted at *p *< 0.05. The SPSS 14.0 program for Windows was used to perform the statistical analysis. All susceptibility experiments were run in duplicate and growth and radioactivity assays in triplicate.

## Authors' contributions

SCO and RA designed the study, performed the cytometric and radioactivity assays and wrote the manuscript. ASD performed growth and susceptibility assays. CPV and AGR also helped in the design of the study and draft the manuscript.

All authors read and approved the final manuscript.
